# Epidemiology and prognosis factors in open globe injuries in the Federal District of Brazil

**DOI:** 10.1186/s12886-021-02183-z

**Published:** 2022-03-09

**Authors:** Marina Berquó Peleja, Felipe Bruno Santos da Cunha, Mariana Berquó Peleja, Juliana Tessari Dias Rohr

**Affiliations:** 1 Hospital de Base do Distrito Federal, SMHS – Área Especial, quadra 101, Asa Sul, Brasília, Distrito Federal, Brasília, CEP 70330-150 Brazil; 2 Centro Universitário de Brasília, Quadra 707/907, Campus Universitário, Asa Norte, Distrito Federal, Brasília, CEP 70790-075 Brazil; 3 Universidade Federal de Goiás, Rua 235, s/n, Setor Leste Universitário, Goiânia, Goiás, CEP 74605-050 Brazil

**Keywords:** Open globe injury, Eye surgery, Traumatic injury

## Abstract

**Objective:**

To identify the epidemiological profile and prognostic factors of open globe injuries that require emergency surgical treatment.

**Design:**

Retrospective cohort study.

**Subjects:**

Patients with OGI who underwent publicly funded emergency surgical treatment in the Federal District from 2014 to 2018.

**Methods:**

Data were collected by reviewing electronic medical records through a questionnaire and tabulated. The statistical analysis was performed in SPSS Statistics 26.0.0.0 (*p* ≤ 0.05).

**Results:**

A total of 359 records were included, corresponding to 336 eyes of 334 patients (294 males and 40 females). The average age was 32.7 years. The affected eye was the right eye in 165 cases, the left eye in 166 cases, and both eyes in 3 cases. The average time between injury and hospitalization was 75.7 h, and the time between injury and surgery averaged 173.7 h. The injury types were as follows: 197 penetrating; 109 rupture; 19 IOFB; 11 perforating. The injuries were in the following zones: 181 zone I; 82 zone II; 70 zone III. The OTS grades were as follows: 57 were classified as grade 1; 101 were grade 2; 142 were grade 3; 28 were grade 4; and 8 were 5. The most commonly performed surgeries were corneal suture, corneoscleral suture, and evisceration. The most common clinical features were traumatic cataract, herniated iris and hyphema. The following were risk factors for poor prognosis: zone III, time between trauma and surgery > 72 h, rupture injury, retinal detachment, disorganization of the eyeball, endophthalmitis, uveal prolapse, OTS classification 1 or 2, and low initial visual acuity. The following factors predicted a good prognosis: initial VA > 1/200, penetrating injury, OTS 4 and zone II.

**Conclusions:**

The high frequency of many of these factors may explain the high rate of severe visual loss found. Injury localization in zone II was identified as a previously unrecognized protective factor against severe visual loss.

## Introduction

Ocular globe trauma is associated with several trauma mechanisms and forces involved, generating different types of injuries that are divided into open and closed. Open lesions result from the involvement of the entire thickness of the corneoscleral ocular structure. Closed injuries are those in which the corneoscleral eye structure remains intact [1–5].

The Birmingham Eye Trauma Terminology (BETT) system standardizes the names of mechanical injuries in the eye, connecting terminologies. Thus, open eye trauma is classified as rupture, referring to full-thickness injury of the globe by trauma with a blunt object, and laceration, when there is full-thickness injury of the globe caused by a sharp object. In addition, lacerations are subdivided into penetrating wounds, intraocular foreign bodies (IOFBs) and perforating wounds. The penetrating wound is marked by a simple laceration, whereas the IOFB is when the foreign object retained causes an entrance laceration. The perforating wound shows two full-thickness wounds on the eye structure, showing the entry and exit of the cutting object [6, 7].

Another classification, the Ocular Trauma Score (OTS), estimates the visual prognosis from clinical data such as initial visual acuity (VA), wound location and the relative afferent pupillary defect (DPAR). OTS ranges from 1 (most severe injury and worst prognosis at 6 months of follow-up) to 5 (less severe injury and better prognosis) [8–12].

### The reality of the Federal District

The Federal District is a Federative Unit of Brazil that does not have municipalities; it is divided into 31 administrative regions. According to the last demographic census conducted in 2010, the population of the Federal District totaled 2.570.160 people [13].

The Integrated Development Region of the Federal District and Surroundings (RIDE) was ruled by Complementary Laws 94/1998 and 163/2018 to facilitate the articulation of administrative actions between the Union, the States of Goiás, Minas Gerais and the Federal District [14].

In the public network of the Federal District and the surrounding region, all patients who require emergency ophthalmic surgery due to laceration of the eyeball are referred to the Base Hospital of the Federal District by the guidelines of the Urgency and Emergency Protocols of the Federal District Health Department since 2006 [15]. Thus, the results obtained from sampling extracted from this hospital reflect the reality of the RIDE.

## Methodology

### Outline

This was a retrospective cohort study to determine the epidemiology and prognostic factors of open globe injuries (OGIs) that underwent emergency surgical treatment in a public tertiary hospital in the Federal District from 2014 to 2018.

The data were collected by reviewing electronic medical records and tabulating them in Microsoft® Excel for Mac, version 16.19. The statistical analysis was performed in SPSS Statistics 26.0.0.0 using the chi-square test, Fisher’s exact test and relative risk (RR), with *p* ≤ 0.05 as the criterion for statistical significance.

The chi-square test was performed for all variables, and those with more than 20% of cells with an expected count < 5 were also subjected to Fisher’s exact test. Those with a total count of 1 have not been tested. Factors for which the chi-square test showed a cell count of less than 5 above 20% were tested separately.

Relative risk was calculated only if the numerator ≠ 0.

### Inclusion criteria

Patient victims of traumatic OGI underwent emergency surgical treatment in a public tertiary hospital in the Federal District, with injury and surgical procedures ranging from 01/01/2014 to 12/31/2018.

### Exclusion criteria

Nontraumatic globe injury; traumatic closed globe injury; cases occurring outside the stipulated period; without minimal information in the medical records (mechanism of injury, date of injury, affected eye, date of surgery, initial visual acuity and after surgical procedure, type of surgery performed).

### Variables

Independents: the origin (city and state), accident environment (home; work; traffic; others), age group (under 18 years; 18–39 years; 40–59 years; 60 years or older), biological sex (male; female), affected eye (right; left; both), type of injury (penetrating; rupture; IOFB; perforating), initial visual acuity (≥ 20/40; 20/50 to 20/200; 19/200 to 1/200; luminous perception (LP) or hand movement (HM); absence of light perception (ALP)), last visual acuity registered in the medical record after surgery (≥ 20/40; 20/50 to 20/200; 19/200 to 1/200; LP or HM; ALP), relative afferent pupillary defect (absent; present; not registered – NR), injury zone (zones: I – restricted to the cornea and limbus; II – from the limbus to 5 mm of surrounding sclera; and III – more than 5 mm from the limbus in the posterior direction along the sclera), time elapsed until hospitalization (≤12 h; > 12 to ≤24 h; > 24 to ≤72 h; > 72 h), time elapsed between trauma and surgery (≤12 h; > 12 to ≤24 h; > 24 to ≤72 h; > 72 h), surgery performed, associated clinical characteristics and calculation of OTS (1 to 5).

Dependent: visual loss, which was categorized as mild (last VA ≥ 20/200) or severe (last VA < 20/200).

Visual acuity was measured with a Snellen chart.

## Results

**Fig. 1:**
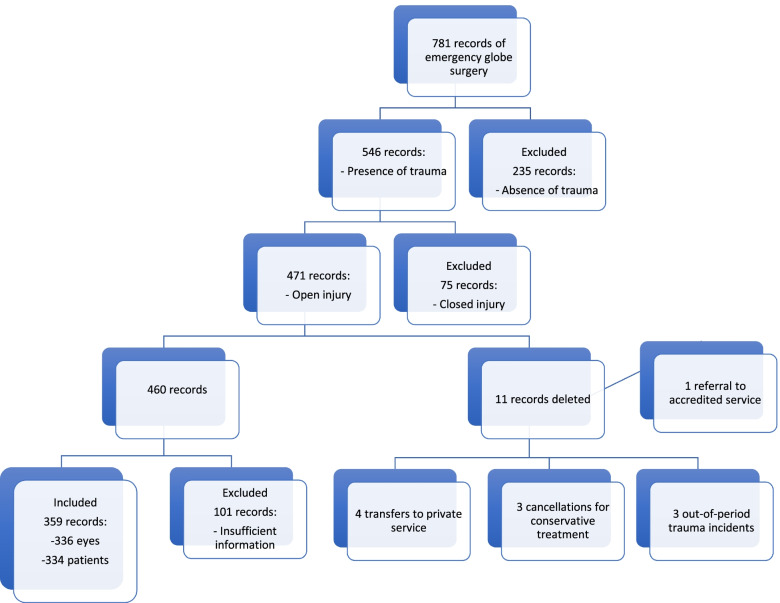
Flow chart of case selection

A total of 781 records of urgent/emergency ophthalmologic surgeries were identified during the research period (2014 to 2018). Inclusion and exclusion criteria were applied (Fig. 1).

The final sample consisted of 359 records of surgeries, referring to 336 eyes and 334 patients, that were included in this study. Three patients had OGI in both eyes, but in one case, surgery was required in only one eye, since the lesion in the other eye was self-sealing and was treated conservatively. Twenty-three records were reoperations.

### Demographic evaluation

The following origin distribution was found: 202 (60.48%) Federal District, 94 (28.14%) Goiás, 17 (5.09%) Bahia, 17 (5.09%) Minas Gerais, 1 (0.30%) Pernambuco, 1 (0.30%) São Paulo and 2 (0.60%) NR. A total of 291 patients came from RIDE.

In the Federal District, the most common origins were Planaltina (31 patients), Ceilândia (23), Sobradinho (20 patients), Gama (17), Samambaia (16) and Brasília (14).

### Epidemiological evaluation

We found 294 (88.0%) males and 40 (12.0%) females in the records.

The average age was 32.74 years old, with extremes of 3 and 80 years and a median of 33 years. By age group, 84 patients (25.1%) were 18 years or younger, 131 (39.2%) were between 19 and 39 years, 94 (28.1%) were between 40 and 59 years and 25 (7.5%) were 60 years or older.

Regarding the injury environment, in 265 (79.3%) NRs, 29 (8.7%) occurred in traffic, 17 (5.1%) at work, 16 (4.8) %) at home, and 7 (2.1%) at other locations.

Analyzing the number of patients, 165 (49.4%) had the right eye affected, 166 (49.7%) had the left eye affected and 3 (0.9%) had both eyes affected. Two of the three bilateral cases occurred in traffic accidents.

The average time between injury and hospitalization was 75.7 h, but the median was 9 h. The average time between injury and surgery was 173.7 h, and the median was 72 h.

Regarding the frequency of the type of injury, 197 (58.6%) were penetrating, 109 (32.4%) were ruptures, 19 (5.7%) were IOFBs and 11 (3.3%) were perforating.

Regarding the afferent pupillary defect, the result was unreported in 314 cases (93.5%), negative in 16 (4.8%), and positive in 6 (1.8%).

As for the zone of ​​the injury, 181 (53.9%) were zone I, 82 (24.4%) were zone II, 70 (20.8%) were zone III and 3 (0.9%) NR.

The initial VA values had the following frequencies: 66 (19.6%) ALP; 178 (53.0%) luminous perception (LP) or hand movement (HM); 52 (15.5%) between 1/200 and 19/200; 34 (10.1%) between 20/200 and 20/50; and 6 (1.8%) with 20/40 or more.

Regarding final VA, the average time between surgery and measurement was 223 days, and the results were as follows: 114 (33.9%) ALP; 96 (28.6%) LP or HM; 58 (17.3%) between 1/200 and 19/200; 36 (10.7%) between 20/200 and 20/50; and 32 (9.5%) with 20/40 or more.

Table 1 describes the performed procedures in descending order of frequency, and Table 2 describes the associated clinical characteristics, whether pre-existing or after the trauma.

**Table 1:** List of surgeries performed, with respective frequencies

Surgery	Count (*n* = 543)	Percentage (%)
Corneal suture	145	26.7
Corneoscleral suture	69	12.7
Evisceration	64	11.8
Scleral suture	53	9.8
Anterior chamber wash without antibiotics	34	6.3
Phakectomy or phacoemulsification without IOL	28	5.2
Vitrectomy	28	5.2
Iridectomy	24	4.4
Eyelid suture	20	3.7
Mass aspiration	12	2.2
Others	66	12.2

*Legend*: *IOL* intraocular lens, *IOFB* intraocular foreign body

**Table 2:** List of clinical characteristics and correlation with visual losses

Characteristic	Count	Incidence/ Prevalence (*n* = 336)	Visual Loss		Chi-square Test (*p*-value)	Fisher’s Exact Test (*p*-value)
			Mild	Severe		
Traumatic cataract	110	32.7%	17	93	0.108	–
Iris herniation	116	34.5%	29	87	0.156	–
Hyphema	115	34.2%	18	97	0.110	–
Vitreous prolapse	77	22.9%	19	58	0.306	–
Uveal prolapse	62	18.4%	7	55	0.046	–
Vitreous hemorrhage	42	12.5%	8	34	0.799	–
Eyelid laceration	32	9.5%	5	27	0.470	–
Disorganization of the eyeball	28	8.3%	0	28	0.005	–
Retinal detachment	24	7.1%	0	24	0.010	0.007
Orbital fracture	22	6.5%	1	21	0.055	0.058
Rupture of the anterior lens capsule	22	6.5%	4	18	0.777	> 0.999
Masses in anterior chamber	16	4.8%	1	15	0.147	0.209
Hypopyon	19	5.6%	1	18	0.090	0.140
Endophthalmitis	19	5.6%	0	19	0.023	0.017
Extrusion of intraocular content	14	4.2%	0	14	0.052	0.083
Iridodialysis	11	3.3%	1	10	0.339	0.472
Luxation of crystalline lens	10	3.0%	1	9	0.402	0.694
Clutter of anterior chamber	10	3.0%	2	8	0.966	> 0.999
Rupture of posterior lens capsule	9	2.7%	0	9	0.122	0.213
Corneal ulcer	6	1.8%	0	6	0.209	0.353
Subluxation of crystalline lens	6	1.8%	1	5	0.813	> 0.999
Eye abscess	5	1.5%	0	5	0.252	0.588
Cellulitis	5	1.5%	1	4	0.976	> 0.999
Corneal foreign body	4	2.0%	2	2	–	0.188
Previous low visual acuity	4	2.0%	0	4	–	0.585
Iris laceration	4	2.0%	0	4	–	0.585
Proptosis	4	2.0%	1	3	–	> 0.999
Previous corneal transplant	4	2.0%	0	4	–	0.585
Lacrimal canal laceration	2	0.6%	0	2	–	> 0.999
Corneal suture dehiscence	2	0.6%	0	2	–	> 0.999
Conjunctival suture dehiscence	2	0.6%	2	0	–	0.042
Intraorbital foreign body	2	0.6%	0	2	–	> 0.999
Corneal transplant dehiscence	2	0.6%	0	2	–	> 0.999
Choroidal detachment	2	0.6%	0	2	–	> 0.999
Choroid thickening	2	0.6%	0	2	–	> 0.999
Secondary glaucoma	2	0.6%	0	2	–	> 0.999
Mass in vitreous	2	0.6%	0	2	–	> 0.999
Corneal tissue loss	2	0.6%	0	2	–	> 0.999
Polytrauma	2	0.6%	0	2	–	> 0.999
Retinal prolapse	2	0.6%	0	2	–	> 0.999
Cranioencephalic trauma	2	0.6%	1	1	–	0.369
Traumatic uveitis	2	0.6%	0	2	–	> 0.999
Infectious keratitis	1	0.3%	0	1	–	–
Cyclodialysis	1	0.3%	0	1	–	–
Conjunctival granuloma	1	0.3%	1	0	–	–
Retrobulbar hematoma	1	0.3%	1	0	–	–
Subchoroidal hematoma	1	0.3%	1	0	–	–
Laceration of muscle upper oblique	1	0.3%	1	0	–	–
Traumatic maculopathy	1	0.3%	0	1	–	–
Megalocornea	1	0.3%	0	1	–	–
Shotgun pellet in face	1	0.3%	0	1	–	–
Retinal rupture	1	0.3%	1	0	–	–
Sepsis	1	0.3%	0	1	–	–
Previous trabeculectomy	1	0.3%	0	1	–	–

When analyzing the distribution according to OTS grade, 57 (17,0%) are classified as 1, 101 (30.1%) as 2, 142 (42.3%) as 3, 28 (8.3%) as 4 and 8 (2.4%) as 5.

### Visual loss and associated factors

After treatment, 137 (40.8%) of the eyes maintained their VA, 96 (28.6%) worsened and 103 (30.7%) improved. Of the total number of records, 68 (20.2%) presented mild loss, and 268 (79.8%) presented severe loss.

Table 3 shows the results of the association tests between the independent variables and visual loss and the relative risk of severe visual loss.

**Table 3:** Association tests between the independent variables and visual loss, and the relative risk of severe visual loss for the significant associations (*p* ≤ 0.05)

Factors	*p*-value (χ^2^)	RR	CI (95%)
Age group (*n* = 334)	0.148	–	–
Biological sex (*n* = 334)	0.232	–	–
RAPD (*n* = 336)	0.266^F^	–	–
Disorganization of the eyeball (*n* = 336)	0.005	1.289	1.214–1.368
Endophthalmitis (*n* = 336)	0.017^F^	1.278	1.206–1.355
Retinal detachment (*n* = 336)	0.007^F^	1.284	1.210–1.362
Uveal Prolapse (*n* = 336)	0.046	1.147	1.028–1.279
Initial VA: ALP (*n* = 336)	< 0.001	1.337	1.247–1.432
Initial VA: LP or HM (*n* = 336)	< 0.001	1.275	1.136–1.431
Initial VA: 1/200 to 19/200 (*n* = 336)	< 0.001	0.698	0.554–0.881
Initial VA: 20/200 to 20/50 (*n* – 336)	< 0.001	0.468	0.306–0.717
Initial VA: ≥ 20/40 (*n* = 336)	< 0.001^F^	a	–
Initial VA: ≥ 20/200 (*n* = 336)	< 0.001	0.400	0.257–0.623
Injury: Penetrating (*n* = 336)	0.002	0.844	0.762–0.936
Injury: Rupture (*n* = 336)	0.019	1.144	1.032–1.267
Injury: Perforating (*n* = 336)	0.129^F^	–	–
Injury: IOFB (*n* = 336)	0.775^F^	–	–
Injury-to-hospitalization time (*n* = 334)	0.951	–	–
OTS 1 (*n* = 336)	< 0.001	1.293	1.200–1.393
OTS 2 (*n* = 336)	0.001	1.216	1.105–1.339
OTS 3 (*n* = 336)	0.148	0.922	0.824–1.032
OTS 4 (*n* = 336)	< 0.001	0.426	0.259–0.702
OTS 5 (*n* = 336)	0.001^F^	0.308	0.093–1.025
Surgery ≤12 h from trauma (*n* = 334)	0.033^F^	0.532	0.226–1.254
Surgery > 12 to ≤24 h from trauma (*n* = 334)	0.125	–	–
Surgery > 24 to ≤72 h from trauma (*n* = 334)	0.277	–	–
Surgery > 72 h from trauma (*n* = 334)	0.010	1.154	1.040–1.279
Zone 1 (*n* = 336)	0.920	–	–
Zone 2 (*n* = 336)	0.001	0.800	0.681–0.940
Zone 3 (*n* = 336)	0.001	1.242	1.136–1.357

*Legend*: χ^2^ = Pearson’s chi-square test, *RR* relative risk, *CI* confidence interval, *RAPD* relative afferent pupillary defect, *VA* visual acuity, *ALP* absence of light perception, *LP* light perception, *HM* movement of hands, *IOFB* intraocular foreign body, *OTS* Ocular Trauma Score, *F* corrected by Fisher’s exact test

^a^numerator = 0, RR could not be calculated

For biological sex, age group, DPAR and injury-to-hospitalization time, the tests showed no significant association with visual loss.

The types of injuries were tested one by one. Severe visual loss was shown to be significantly increased in rupture (*p* = 0.019) and penetrating trauma (*p* = 0.002). The perforating and IOFB types showed no association with visual loss.

Table 2 shows the correlation between associated clinical characteristics and visual loss. Retinal detachment, disorganization of the eyeball, endophthalmitis and uveal prolapse were shown to be statistically related to visual loss.

In the evaluation of the injury zone, a significant association with visual loss was noted by the chi-square test (*p* <  0.001), but this occurred only for zones II and III. Zone II was a protective factor, and zone III was a risk factor. Zone I showed no significant difference in severe and mild visual loss.

The OTS classification showed a statistical association with visual loss (*p* <  0.001). Subgroups 1 and 2 were risk factors, and subgroup 4 was a protective factor for severe visual loss. Subgroups 3 and 5 showed no significant difference.

The chi-square test between initial VA and visual loss showed that there was a significant association (p <  0.001). The ALP and LP/HM subgroups had an increased risk of severe visual loss, and patients with an initial VA ≥1/200 had a reduced risk.

Regarding injury-to-surgery time, the subgroups “≤12 h”, “> 12 to ≤ 24 h” and “> 24 to ≤ 72 h” showed no significant association with severe visual loss. The “> 72 h” subgroup had a significant association, increasing in the probability of severe visual loss.

## Discussion

OGI is a widely studied topic; however, there are few statistical data on its epidemiology in the Federal District [16, 17].

Rohr et al. (2016) identified the profile of pediatric globe injury in children under 15 who attended the Base Hospital in the Federal District between June 2012 and January 2013, of which only 20% had OGI (*n* = 103) [7]. Vieira (2007) outlined the profile of 2844 patients treated during September 2003 at the same hospital, identifying that 62% of the patients were male, 17% were from other states, and 30% of the patients were due to eye injury [18]. None of the two studies had the same focus as the present study, but they indicate the high frequency of eye injury in DF.

In the five years included in the survey, practically 70% of urgent/emergency ophthalmologic surgeries in the public network of the Federal District were due to eye injury, with the majority of these types of injuries being open (86.26%), which had an average of 93 cases per year. This corresponds to an incidence of 3.6/100,000 inhabitants, considering the DF population in the 2010 census. This result is in line with other publications that point to a higher frequency of surgery in OGI [19–23]. A Chinese study (*n* = 2009) in the period from 2010 to 2014 attributed 70.7% of eye injuries to OGI [22]. A ten-year study (2005–2014) in New Zealand including OGI that underwent surgical repair calculated an average incidence rate of 2.8/100,000 inhabitants, another in Turkey (2009–2013) found 3.5/100,000 per year and one in Israel (1996–2005) 3.1/100,000 inhabitants [24–26]. Compared to the New Zealand and Israel study, the incidence in DF was higher but very similar to that found in Turkey.

For every 10 cases, 4 were from other states, mainly Goiás. This represents almost twice as much as that found in a previous study at the same hospital that evaluated emergency room visits [18]. In 87.1% of the cases, the patients came from the RIDE.

There was an evident predominance in men, 88% of cases. These data are in line with several studies on ocular trauma, whether in general or specifically OGI, with a male frequency ranging from 73.3 to 90% [5, 9, 19–25, 27–35].

Approximately 67% of the patients were between 19 and 59 years old, with an average age of 32.7 years. In the literature, this average ranges from 30 to 38.3 years [5, 20, 35]. The exclusion of records that did not report visual acuity probably led to the exclusion of many neonates and preverbal children, in whom this measure is more difficult or impracticable.

The most affected age group is economically active, generally requiring time off work. In addition to their treatment expenses, these people stop producing. This shows the importance of reducing the time of care and hospitalization [22].

The frequency was similar between the two eyes, and injuries were rarely bilateral; these results were consistent with the findings of several prior studies, where the difference in laterality was no more than 10% [20, 24, 25, 27, 34, 36].

The lack of information about the trauma environment and DPAR in most of the records makes it impossible to correctly assess the distribution of these variables. In the literature, the main accident environments are work, traffic and home [37–39].

In Saudi Arabia, a similar study shows a penetrating type in 37.5% of cases; rupture by 32.5%; perforation in 26.7%, and IOFB in 3.3% [32]. In Israel, the frequency was IOFB in 38.1%; rupture by 28.9%; penetration in 27.1% and perforation in 5.9% [26]. In Turkey there is work showing penetration in 75% of cases, rupture in 13%, IOFB in 8%, and perforation in 4% [34]. In Portugal, penetration occurred in 48.9%, rupture at 31.9%, IOFB at 13.2%, and perforation at 2.2% [33]. In New Zealand, 56.4% penetrating; 35.6% rupture; 7.3% IOFB and 0.8% perforation [24]. In an Australian study, globe rupture and penetration were the most common injuries [30]. Except in the Israeli study, there was a tendency for greater involvement of the penetration and rupture types, as we found.

More than half of the lesions affected zone I, consistent with the literature, where it is the most frequently affected zone, between 38.1 and 53% [24–26, 32, 34–36].

The average time that is taken from trauma to hospital and surgery was above those found in other studies, which was between 4.9 h to 1.3 days and 5 h to 39,9 h, respectively, with 72.8–94.8% of the patients hospitalized within 24 h of the trauma [24, 25, 29, 33, 36, 40]. However, the median is a better evaluation parameter since the variables in question do not present a normal distribution and have extreme values [41]. The median values ​​found were reasonable, but the time to surgery was still above the average of most studies.

Regarding the initial VA, the majority had LP/HM, which is very similar to that found in the literature [24, 25, 28, 31, 35, 40].

The most common final VA was ALP. The literature shows a wide range of variation in these result, with one study reporting that 65.6% had VA ≥ 20/200 and another reporting a predominance of ALP [25, 33]. Rao et al. (2010) showed that after 6 months, 34.8% of the patients had ≥20/70 vision, and the same number had < 20/400 [35]. In Saudi Arabia, 40.8% had vision between 20/200 and 20/50 [32]. In New Zealand, 46% of patients had vision ≥20/40 at the end of treatment [24].

Previous studies have observed VA improvement after treatment in most cases, varying between 55.7 and 58% of patients, while in our records, the majority had parameter maintenance [5, 26, 35].

We found that more than 70% of the traumas were classified as OTS 3 and 2, tending to be of medium to severe severity. A South African study had similar results, with 66.86% in these categories [27]. Ozturk et al. (2019) found a majority in 2 (45.2%) and 1 (33%) [36].

The most performed surgeries, in decreasing order, were corneal suture, corneoscleral suture, evisceration, scleral suture, and anterior chamber washing. There is a convergence that primary repair is the most frequent, followed by evisceration [5, 27].

The main associated clinical characteristics, in decreasing order, were traumatic cataract, iris hernia, hyphema, vitreous prolapse and uveal prolapse. There is a difference in the literature because of the high incidence of traumatic cataracts. Atik et al. (2018) found hyphema, herniated iris, vitreous hemorrhage, eyelid/eyebrow laceration, and traumatic cataracts as the most frequent cataracts [25]. Rao et al. (2010) reported herniated iris, hyphema, traumatic cataract, vitreous hemorrhage and endophthalmitis [35]. In a Saudi Arab study, the main ones were iris lesions, hyphema, vitreous hemorrhage, aphakia, and retinal damage [32].

The severe visual loss found (79.8%) was above that described in the literature. Atik et al. (2018) observed its occurrence in 65.6% of cases in a Turkish hospital [25]. Teixeira et al. (2014), in a Portuguese study, observed 60% after 6 months of trauma [33]. In New Zealand, Israel, and Saudi Arabia, this rate was much lower, 39.3, 40, and 45.9%, respectively [24, 26, 32].

We found a significant association with severe visual loss in lesion in zone III, time between trauma and surgery > 72 h, rupture injury, retinal detachment, disorganization of the eyeball, endophthalmitis, uveal prolapse, OTS classification 1 and 2, initial visual acuity ALP, LP or HM. Such results reaffirm what was found in previous research, which also relate the presence of relative afferent pupillary defect, extensive wound, eyelid laceration, hyphema, damage to the lens, vitreous prolapse and vitreous hemorrhage [5, 6–9, 27, 42–46]. The high frequency of these characteristics may explain why severe visual loss was found in almost 80% of cases. This also reaffirms the medium to high severity of this type of injury, indicated by the OTS calculation.

Initial VA > 1/200, penetrating injury, OTS 4 and zone II showed an inverse association with severe visual loss. In the literature, only zone II is not described as a protective factor. Other known protective factors are OTS 3, injury in zone I, pediatric age, and injury restricted to the anterior segment [26, 33–35].

Knowledge of prognostic factors enables specific and targeted actions aimed at reducing visual loss due to open eye trauma. Most factors are related to the severity and characteristics of the trauma, which could be improved by educational actions and guidance on the use of personal protective equipment in the work environment and in recreational activities [18].

In Brazil, Regulatory Norm number 6 establishes that companies must have their occupational risks estimated by a trained professional and provide all necessary safety equipment to their employees free of charge to avoid work accidents. It is up to the government to encourage and supervise correct compliance with the standard [47].

Another possible prognostic factor that could be modified is the time between trauma and surgery, which should be prioritized so that it occurs before 72 h. For this purpose, the community must be well informed about the points where emergency eye care is available. The Unified Health System is universal and free in Brazil; therefore, financial issues do not represent a problem for the user. Another point that could be improved is the duration of surgery after hospitalization. Surgeries are prioritized to take place according to the severity of the injury and risk of death or loss of function. The long surgical delays demonstrate the high demand for urgent/emergency surgeries, which could be resolved by expanding existing services and opening new care/surgery units. Another option would be the creation of an exclusive ophthalmology service, which would have more agility in performing these surgeries and addressing other ophthalmological urgencies/emergencies [48].

## Conclusion

There have been several studies reviewing the epidemiology of open globe injury in different locations; these studies are in agreement that there is a predominance of males, young adults, and patients with low initial VA. The high morbidity of this type of injury emphasizes the importance of the use of personal protective equipment and the adoption of legislative regulations for eye safety, reducing the impact of globe injuries on the community [49–51].

Our research came up with similar results found in the literature, but with a greater divergence regarding the time between trauma and hospitalization/surgery, which can be improved, and we found Zone II as a protective factor for severe loss. The high frequency of poor prognosis factors may explain the large severe visual loss in the studied population.

The demographic data generated in this work can help health entities develop assistance strategies for victims of globe injury, as well as education and prevention actions and improvements in ophthalmological services.

Our study is limited by its retrospective nature and the absence of certain data from the medical records evaluated.

## Data Availability

Data were collected by reviewing electronic medical records based on the list of ocular emergency surgeries performed in the Hospital de Base do Distrito Federal from 01/01/2014 to 31/12/2018. The datasets used and/or analyzed during the current study are available from the corresponding author on reasonable request.
